# Characteristics and Outcome of Surgically Treated Patients with Intradural Extra- and Intramedullary Spinal Metastasis—A Single-Center Retrospective Case Series and Review †

**DOI:** 10.3390/curroncol31070304

**Published:** 2024-07-19

**Authors:** Hanna Veronika Salvotti, Alexander Lein, Martin Proescholdt, Nils-Ole Schmidt, Sebastian Siller

**Affiliations:** Department of Neurosurgery, University Hospital, University of Regensburg, Franz-Josef-Strauss-Allee 11, 93053 Regensburg, Germanymartin.proescholdt@klinik.uni-regensburg.de (M.P.);

**Keywords:** metastasis, intradural, intramedullary, extramedullary, outcome, survival

## Abstract

Objective: Intradural spinal metastases are considered rare. At present, limited information is available on incidence, surgical management, and outcomes. Methods: We conducted a retrospective patient chart review from 2002 to 2024, identifying all patients surgically treated for intradural spinal metastases. Clinical, surgical and survival data were collected and compared to literature data for patients surgically treated for extradural spinal metastases. Results: A total of 172 patients with spinal metastases were identified with 13 patients meeting inclusion criteria (7.6%). The mean age at diagnosis of intradural spinal metastases was 52 ± 22 years, with diverse primaries including lung (*n* = 3), breast (*n* = 2), sarcoma (*n* = 2), and six unique entities. Intradural spinal metastasis was diagnosed on average of 3.3 years after primary diagnosis. In total, we observed five (38%) intradural-extramedullary and eight (62%) intramedullary metastases, located in the cervical (38.5%), thoracic (46.1%) and lumbar spine (15.4%). The most common preoperative symptoms were pain, sensory changes, and gait ataxia (each 76.9%). Gross total resection was achieved in 54%, and local tumor control in 85%. Postoperatively, 92% exhibited clinical improvement or stability. Most frequent adjuvant treatment was radio- and/or chemotherapy in 85%. The average survival after operation for spinal intradural metastases was 5 months, ranging from 1 month to 120 months. The location of the intradural metastasis in the cervical spine was associated with a significantly more favorable survival outcome (compared to thoracic/lumbar location, *p* = 0.02). Conclusions: Intradural location of spinal metastases is rare (7.6%). Even so, surgical resection is safe and effective for neurological improvement, and survival appears lower compared to the reported survival of extradural spinal metastases.

## 1. Introduction

Intradural extramedullary spinal metastasis represent a rare entity of systemic oncological diseases accounting for approximately 5–10% of all intraspinal metastatic lesions [1,2,3]. According to the literature, intradural intramedullary metastases are even more infrequent entities, accounting for 0.9–2.1% of all secondary malignancies [4,5].

Due to innovation in diagnostic tools as well as improvement of treatment options in oncological patients with longer overall survival, a slow increase in the incidence of this type of spinal metastases seems to be observed in the past years [6,7,8]. While extradural spinal metastases are frequently addressed in the literature, studies on the incidence and therapy of intradural metastases are still rare, and single case reports dominate in the literature [9]. Cancer entities most frequently mentioned in the literature for intradural metastases include lung carcinoma, mamma carcinoma, brain tumors, and prostate carcinoma [8,10], while treatment options described in historical case reports remain highly controversial, and range from surgical resection to chemotherapy, radiosurgery, and/or radiotherapy [11,12,13,14]. Limited evidence is available that surgery in patients with intradural metastases might improve survival compared to conservative treatment methods [15,16].

Per se, the prognosis of spinal metastasis in general is, even today, poor, as they usually occur in advanced cancer stages. An average survival of approximately 12 months is reported in the literature [17]. Little so far is known if this is also true for the rare type of intradural spinal metastases, or if survival differs for this subtype of spinal metastases, especially due to the fact that intradural metastases often lead to a more rapid progression of physical impairment and a consequent reduction in quality of life and fitness for oncological adjuvant treatment [12].

The aim of this study was to determine the incidence, clinical, and pathological features, as well as post-operative outcome and survival of extra- and intramedullary intradural metastases of systemic malignant tumors treated surgically at our institution.

## 2. Materials and Methods

### 2.1. Study Design, Patient Selection, and Participation

We conducted a single-center retrospective chart review at our tertiary academic reference center including all patients undergoing surgical treatment for a spinal metastasis of a systemic malignant neoplasm between April of 2002 and March of 2024 at the Department of Neurosurgery of the University Hospital Regensburg, Germany. Patients with an intradural extra- or intramedullary metastasis were identified, and data regarding demographics, primary cancer histopathology, spinal location and relation to the spinal cord, clinical course, pre- and postoperative neurological status, extent of tumor resection, oncological treatment regime, and long-term follow-up were acquired from the patients’ records available at our institution, as well as from the University Clinical Cancer Registry at the University Hospital Regensburg.

Patients who underwent surgery for an intradural CNS-inherent tumor (e.g., tumor entities listed in the 2021 WHO Classification of CNS tumors) in the spinal region were excluded.

This study was conducted according to the principles expressed in the Declaration of Helsinki. The Ethical Committee of the University of Regensburg approved the study (No. 23-3588-103).

### 2.2. Clinical Evaluation

Detailed neurological assessment, as well as classification of the functional status using the McCormick scale [18], was performed in all patients with intradural metastases preoperatively at admission for planned surgical treatment, at discharge after surgery, and at every follow-up timepoint. Clinical follow-up of patients was initially performed every 3 months and until the timepoint of death. Detailed neurological examination included motor function assessment, evaluation of sensory function and pain level, as well as gait and vegetative assessment.

### 2.3. Surgical Procedures

Decision for or against surgery was always carried out on the basis of an individual interdisciplinary discussion, with the oncological team integrating the patient’s past treatment history, current status and preferences, the available postoperative adjuvant treatment options, and the potential goals of surgery (e.g., neurological improvement, spinal stabilization, etc.). Metastasis location and spinal level, as well as distribution within the spinal cord, were evaluated based on contrast-enhanced and non-contrast enhanced MR imaging of the spine, which was carried out in all patients preoperatively. Based on intraoperative clinical as well as radiographic observations, tumors were characterized in extramedullary, intramedullary, or extra- and intramedullary location.

Anesthesia for surgery was performed with total intravenous anesthesia, and subsequently all patients were placed in the prone position. For tumor resection, a posterior midline approach was performed, and the lamina and spinous processes overlying the tumor were exposed. Subsequently, hemilaminectomy or laminectomy were performed for tumor resection. If the metastasis did not approach the spinal cord surface to serve as an entry point for intramedullary tumor resection, a midline myelotomy was performed by sharp dissection after visual identification and marking of the anatomical midline by the surgeon. Resection was performed under microscope- and ultrasound-guidance according to the state-of the-art microsurgical techniques, as well as with the continuous multimodal IONM of both somatosensory and motor evoked potentials, as well as free-running electromyography utilizing an integrated IONM system (ISIS, Inomed Co., Emmendingen, Germany) on discretion of the performing surgeon. After resection, a watertight closure of the dura was performed. Adjuvant treatment was indicated and performed on the basis of an interdisciplinary tumor conference decision.

In all cases, tumor tissue obtained intraoperatively was sent to the Department of Pathology of our institution for analysis of the tumor entity. The extent of resection was determined according to the operative records as well as the contrast-enhanced and non-contrast-enhanced MR imaging of the spine at the 3-month follow-up. Gross total tumor resection was defined as complete tumor removal according to the operative records and no residual tumor-suspect contrast-enhancement on a postoperative MRI scan, while removal of >20% (but less than the gross total resection) of the tumor was termed subtotal resection and <20% as biopsy only. Local tumor control was assumed in cases of no further radiological change in the surgical level in further MRI follow-up scans compared to the 3-month follow-up MRI scan, while any tumor-suspect change in contrast-enhancement pattern according to the radiological evaluation was classified as local tumor recurrence/progression.

### 2.4. Statistical Analysis

Statistical analysis was performed using Sigma Plot for Windows v.11 (Systat Software Inc., San Jose, CA, USA). For the comparison of group differences, Student’s *t*-test was used for numeric values, Mann–Whitney Rank Sum test for ordinal variables, and χ^2^-test resp. Fisher’s exact test (in case of 2 × 2-contingency tables) for nominal variables. The reference point of this study was the date of initial diagnosis of the primary cancer (reference point 1) as well as the date of surgery for diagnosis of intradural spinal metastasis (reference point 2) of the individual patient. Patients were followed until death from any cause, or were censored at the day of last follow-up in March 2024 (end point 1). End points were overall survival (reference point 1—end point) and survival after surgery for the intradural spinal metastasis (reference point 2—end point). Survival data were analyzed using the Kaplan–Meier method. Prognostic factors were obtained from proportional hazards models (Cox regression models). *p*-values below 0.05 were considered statistically significant.

## 3. Results

### 3.1. Patients’ and Tumor Characteristics

During our study period, a total of 172 patients with intraspinal metastases were surgically treated in our institute. Out of these 172 patients, 13 patients (13/172 = 7.6%) with intradural spinal metastases were identified, with 5 patients harboring an completely extramedullary and 8 patients an at least partly intramedullary spinal metastasis. One patient with a lumbar extramedullary metastasis also had a small extradural tumor component, suggesting a per continuitatem spread across the dura. Mean age at diagnosis of the intradural metastasis was 52 ± 22 years, and there was a predominance of the male gender (male/female: 1.6/1). Median McCormick score was 3 (range: 2–4), and median KPS was 70 (range: 50–90).

Median time period from primary cancer diagnosis to the diagnosis of the intradural spinal metastasis was 40 months (range 8–102 months); in 2 patients (15%) the clinical manifestation of the intradural spinal metastasis led to the first diagnosis of the primary cancer (lung cancer resp. prostate cancer). While 54% of the patients had evidence of multiple systemic metastases at the timepoint of primary cancer diagnosis, the rate increased to 77% at the timepoint of diagnosis of the intradural metastasis. Seven patients (54%) had evidence of additional central nervous system metastases at the timepoint of diagnosis of the intradural metastasis or during further postoperative follow-up.

Baseline patients’ and tumor characteristics are displayed in Table 1 and Table 2. There was no significant difference in these characteristics between patients harboring completely extramedullary vs. extra-/intramedullary spinal metastases.

### 3.2. Surgical Characteristics and Postoperative Outcome

Two patients (with extramedullary spinal metastases) underwent surgical tumor resection on an emergency basis due to rapidly developing neurological symptoms of spinal cord compression, while 11 patients underwent elective tumor resection. The gross total tumor resection rate was 54%, and the rate of local tumor control was 85%. There were no postoperative surgical complications. Postoperative radiation and/or chemotherapy were the most frequent adjuvant treatment modalities, and were applied in 85% of the patients. One long-term survivor of a systemically metastasized cutaneous adnexal carcinoma underwent three reoperations for local recurrence of an extra-/intramedullary spinal metastasis 1.5, 6, and 15 years after the index operation.

In total, 92% of the patients showed a stable or postoperative improved neurological status after surgery, while one patient suffered from a neurological deterioration. Median postoperative McCormick score was 3 (range: 1–4).

Details for surgical characteristics and postoperative outcome are displayed in Table 3, with no significant differences between patients with completely extramedullary vs. extra-/intramedullary spinal metastases.

### 3.3. Survival Analysis

**Figure 1:**
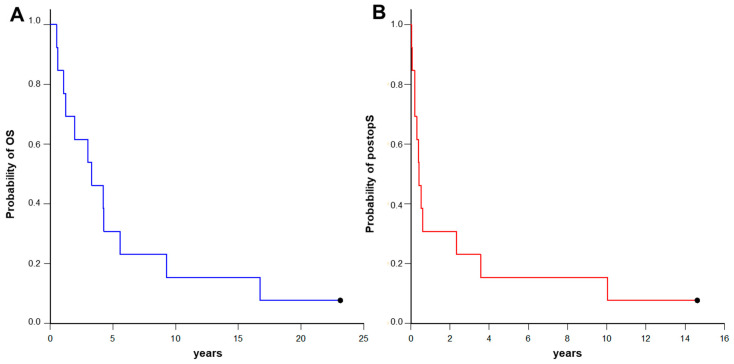
Kaplan-Meier curves for overall survival (OS) after initial diagnosis of the primary cancer (**A**) as well as survival after surgery for intradural spinal metastasis (postopS) (**B**) for 13 patients with intradural spinal metastasis.

At the time of last follow-up (March 2024), 12 patients were deceased (92%). Median OS from diagnosis of primary cancer to death was 39 months (Figure 1A). Death was tumor-related in all patients.

**Figure 2:**
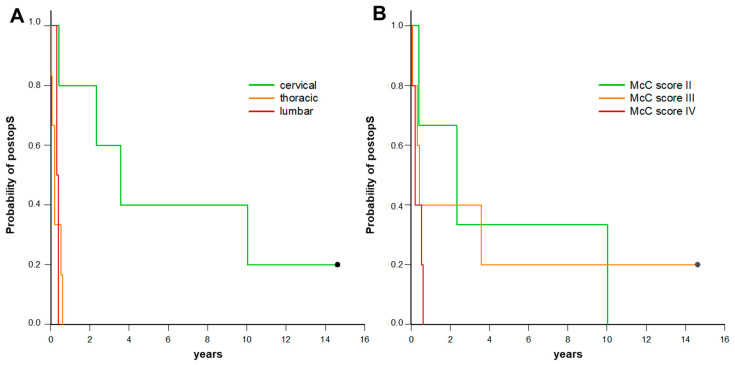
Kaplan-Meier curves survival after surgery for intradural spinal metastasis (postopS) stratified by location of the intradural spinal metastasis (**A**) and by preoperative McCormick score (**B**) for 13 patients with intradural spinal metastasis.

Median survival after surgery for the intradural spinal metastasis was 5 months, ranging from as low as one month to as high as 120 months (Figure 1B). Uni- and multivariate regression analyses for identification of preoperative risk factors affecting survival after surgery for the intradural spinal metastasis are displayed in Table 4. The location of the intradural metastasis in the cervical spine was associated with a more favorable survival outcome (compared to thoracic or lumbar location) both in uni- and multivariate analyses (*p* = 0.02 and *p* = 0.02), while a higher preoperative McCormick score was associated with a poorer survival outcome only in multivariate analyses (*p* = 0.04). Survival after surgery for the intradural spinal metastasis stratified by location of the intradural metastasis and by preoperative McCormick score are displayed in Figure 2. Uni- and multivariate regression analyses for the identification of preoperative risk factors affecting survival after surgery for the intradural spinal metastasis (extent of resection, postoperative McCormick score, postoperative neurological deterioration, adjuvant radio-/chemotherapy) were also performed, but did not show any significance.

## 4. Discussion

Due to the advancement in diagnostic modalities and oncologic treatment methods, the incidence of intraspinal metastases seems to have slowly increased in recent years, and thus might gain further importance in everyday clinical practice in the future [6,7,8]. This is especially true because spinal intradural metastases may cause clinical symptoms such as pain, paresthesia, and neurological deficits, often leading to a severely reduced quality of life [19,20]. Per se, survival prognosis of spinal metastasis in general is, even today, poor, as they usually occur in advanced cancer stages. Little so far is known if survival prognosis might even be worse in the rare type of intradural spinal metastases or not, and if surgical treatment is safe and feasible in patients with intradural spinal metastases.

In our surgical review, we decided to focus on surgically treated intradural intra- and extramedullary metastases and aim to determine the incidence, clinico-pathological features, and clinical/functional as well as survival outcome of intradural spinal metastases of systematic malignant tumors. The incidence of patients with intradural metastases in our case series was of 7.6%. This result is comparable with findings of historical autopsy studies, where intradural extra- or intramedullary metastases were found with an incidence of approximately 5%, and therefore underline the trend in increasing incidences in recent years [3,8,21]. The mean age at presentation was 52 years, comparable to previous case series, and the majority of our patients (77%) had multiple systemic metastases at the time of diagnosis of the intradural spinal metastasis that is also in line with previous publications, where intradural spinal metastases are reported to be found in advanced systemic tumor stages [9,22,23,24].

Despite the advanced stage of tumor disease at the timepoint of diagnosis of the intradural spinal metastasis in the majority of our study patients, surgical treatment was safe and feasible in every single case without surgery-related mortality or systemic morbidity. Gross total tumor resection of the intradural metastasis was achieved in 54% of cases, and in 46%, subtotal resection was achieved; there were no cases of biopsy only in our report. This finding is comparable with those of historical case series by Wostrack et al. and Manzano et al., where total tumor resection was achieved in up to 56% of cases. While in these case series, up to 67% of patients reported improvement/stability of symptoms postoperatively, in our study, over 90% of the included patients experienced a clinical improvement or stable finding postoperatively, as measured using the McCormick Scale [4,18]. These findings indicate that, with advances in microsurgical techniques and perioperative adjuncts (e.g., IONM) over the last decades, surgical treatment of intradural metastases is currently feasible with a good efficiency as well as safety. Similarly to our results, a recent case series by Kritikos et al., including five patients treated surgically for an intramedullary metastasis, found an improved or stable neurological status in all patients after surgery [23].

However, even today, survival prognosis of spinal metastasis in general remains poor. For the classic manifestation of spinal metastases as an extradural tumor, a mean survival of 7.7 to 17 months is reported in the literature [17,25,26,27,28,29,30,31]. Little so far is known if this is also true for the rare type of intradural spinal metastases, or if survival differs for this subtype of spinal metastases. A historical case series of Schick et al. over 20 years ago, comparing intra-and extradural spinal metastases, reported hints for a markedly reduced survival time in patients with intradural in comparison to those with extradural metastases [32]. However, it remains unknown if this might also be true today considering the major advances in diagnostic tools as well as surgical and oncological treatment options in recent decades. Studies reporting on survival outcome of patients with intradural spinal metastases in recent times are still very scarce. We have intensely reviewed the current literature and collected the few case series reporting on survival outcome (see Table 5). The reported mean survival time of intradural spine metastases ranged from as low as 5 to as high as 9.6 months [1,4,6,8,9,23,33,34,35]. However, the significance of those reports is severely limited, especially with regard to a high rate of loss to follow-up or a too-short follow-up time period; the rate of patients needed to be censored from survival analysis ranged as high as 50% in those reports, and therefore markedly limits the validity of those data. In our study, we followed patients’ clinical outcome until timepoint of death (92% of our patients) or for a minimum of 10 years (8% of our patients), making it possible to obtain valid statements about patients’ survival time, which was 5 months in mean. Moreover, in the case series of Sung et al. [34] (*n* = 8), Payer et al. [8] (*n* = 22), and Goyal et al. [35] (*n* = 8), intramedullary metastases were analyzed, including the origin of both secondary malignancies as well as primary CNS tumors (e.g., ependymomas, gliomas, medulloblastomas) which limits the validity and comparability of these case series with those with a focus on true intradural metastases of secondary malignancies. Of note, in our study, a better preoperative McCormick Score was a significant factor for a more favorable survival after intradural spinal metastasis surgery in multivariate analysis, while the location of the intradural metastasis in the cervical spine was associated with a statistically significant more favorable survival (compared to thoracic or lumbar location) both in uni- and multivariate analysis. A possible confounder of this finding could be that, due to more severe symptoms such as gait abnormalities or problems with balance due to myelopathy, cervical spine metastases may be diagnosed at an earlier cancer stage as compared to metastases in the thoracic or lumbar region, and may therefore be associated with a more favorable prognosis. Analysis of other risk factors affecting survival (like extent of tumor, postoperative McCormick score, postoperative neurological deterioration, and adjuvant radio-/chemotherapy) did not show any further significant findings.

### Strengths and Limitations

This study bares several strengths and limitations. This is a study with a long observational period of 10 years and more for each individual patient, making it possible to observe the incidence of intradural spinal metastases over an overall time period of two decades and precisely analyze the survival from diagnosis of primary cancer and surgery for intradural spinal metastasis until death or long-term follow-up. A further strength of our study is represented by the regular follow-ups of patients until the timepoint of death, which makes it also possible to evaluate clinical presentation over time. However, as more than half of the patients in our series additionally had brain metastases at the timepoint of surgery of the intradural spinal metastasis (and brain metastases were progressive in part of the patients during further follow-up), determination of the proportion of neurological and functional changes associated with the lesion at the surgical site compared to other CNS metastases is challenging. Nevertheless, overall, the achieved stabilization or improvement of the neurological status after surgery was maintained in our patients during further follow-up. Moreover, due to the rarity of this tumor entity, the overall patient number is limited; even so, we here present one of the larger recent case series in the literature. Moreover, this is a study of a retrospective and non-randomized nature, limiting the level of evidence. In the future, prospective multidisciplinary studies with a larger patient population are needed to further analyze the benefit of surgery in intradural spine metastases of malignant systemic neoplasms.

## 5. Conclusions

Intra- and extramedullary spinal metastases represent a rare tumor manifestation. They typically occur in advanced tumor stages, and are associated with impairing neurological symptoms and reduced life expectancy. In our study, we analyzed the clinical presentation and outcome of patients with intradural spinal metastases treated at out institution. Our study showed that surgical resection in intradural spinal metastases represents a safe and efficient method for improving clinical outcome. However, overall survival still remains poor with a mean OS of 5 months after diagnosis of the intradural spinal metastasis, which seems to be short compared to the survival times for extradural spinal metastases.

## Figures and Tables

**Table 1:** Baseline patients’ and tumor characteristics at the timepoint of diagnosis of intradural spinal metastasis *.

Characteristics	(*n* = 13)
gender, male/female	5–8 (8/5)
mean age, yrs	52 ± 22
primary cancer, no. (%)	
lung	2 (15.4)
breast	2 (15.4)
sarcoma	2 (15.4)
kidney	1 (7.7)
colorectal	1 (7.7)
cutaneous adnexal	1 (7.7)
melanoma	1 (7.7)
germ cell tumor	1 (7.7)
cancer of unknown primary (CUP) syndrome	2 (15.4) [lung cancer, prostate cancer]
median time between diagnosis of primary cancer and intradural spinal metastasis, months (range)	40 (8–102)
multiple systemic metastases, no. (%)	10 (76.9)
location of intradural metastasis, no. (%)	
cervical	5 (38.5)
thoracic	6 (46.1)
lumbar	2 (15.4)
relationship to spinal cord, no. (%)	
completely extramedullary	5 (38.5)
extra-/intramedullary	6 (46.1)
completely intramedullary	2 (15.4)
extent of tumor, no. (%)	
single-level	9 (69.2)
two-level	3 (21.1)
three-level or more	1 (7.7)
preoperative KPS, median (range)	70 (50–90)
preoperative symptoms, no. (%)	
paresis	6 (53.8)
sensory changes	10 (76.9)
gait ataxia	10 (76.9)
pain	10 (76.9)
bladder/bowel dysfunction	3 (21.1)
non-ambulatory	3 (21.1)
preoperative McCormick Score, no. (%)	
I	0 (0.0)
II	3 (21.1)
III	5 (38.5)
IV	5 (38.4)

* Mean values are presented ± standard deviation.

**Table 2:** Description of cases with intradural metastases.

No.	Sex	Primary Cancer	Initial Manifestation of Primary Cancer	Initial Therapy of Primary Cancer	Time Period between Primary Cancer Diagnosis and Metastasis Diagnosis	Age at Operation	Adjuvant Therapy of Metastasis	Spinal Level of Metastasis	Location of Metastasis	Symptoms of Metastasis	Systemic Metastases	Brain Metastases	Additional Spine Metastases	Postoperative Neurological Outcome at Discharge	Resection Status
1	m	Melanoma	Lentigo malign melanoma, macula	Operative resection	2444	71	Interferon therapy	C2	EM	Left-sided neck pain	Adrenal gland, abdominal wall, lung, retroperitoneal	Yes	No	Pain relieved, no neurological deficits	Gross-total
2	m	Sarcoma	Spinal metastasis	Operative resection	0	38	Chemotherapy, radiotherapy	C 7/T2	EM	Incomplete cross-section, left-sided leg weakness	No	No	No	Improved motor and sensory function, ambulating with aid	Gross-total
3	f	Bone Marrow (AML)	AML	Extern therapy, no information available	1494	11	Radiotherapy	T 6–9	EM	Paraplegia and urinary retention	Bone and soft tissue	No	No	Improved motor function, persisting urinary retention	Subtotal
4	m	Lung (small cell)	Lung carcinoma with dyspnea and cough	Radio-chemotherapy	360	65	Palliative radiotherapy	T 9–10	EM	Lumbar spine pain with radiation to the right leg, hypoesthesia, 4/5 paresis, bladder dysfunction	Mediastinum, adrenal gland	Yes	No	Pain and bladder dysfunction relieved, improved motor and sensory function	Gross-total
5	m	Germinoma	Headache	VCS, chemotherapy	246	18	CP/VP 16 Block	C 3	EM	Neck pain	No	Yes	No	Pain relieved	Gross-total
6	m	Renal (clear cell)	Osseous tumor left scapula	Left side nephrectomy	344	38	Chemotherapy, radiotherapy	L 1–5	IM	Breeches hypoesthesia	Soft tissue	No	Yes	Stable compared to preoperative status	Subtotal
7	m	Lung (large cell)	Spinal metastasis	No therapy	43	74	Radiotherapy	L 1	IM	Back pain, paresthesia in right leg	Lymph nodes	Yes	No	Stable compared to preoperative status	Subtotal
8	f	Breast	Breast carcinoma	Extern therapy, no information available	1203	73	Radiotherapy	T 9–10	IM	Weakness of left > right leg, paresthesia and pain in both legs, not ambulatory	Lung	No	Yes	Pain relieved, improved motor function of left leg, ambulating with aid	Subtotal
9	f	Breast	Breast carcinoma	Operative resection	429	50	Radiotherapy	C1	IM	Vertigo, paresthesia in all extremities	Bone, lymph nodes	Yes	No	Deteriorated (postoperatively new right-sided hemiparesis)	Gross-total
10	f	Sweat glands	Sweat gland carcinoma	Operative resection	2272	48	Radiotherapy	C 6–8	IM	Pain in left arm, paresthesia in left hand, weakness in left arm	Bone	No	No	Pain relieved, improved motor and sensory function	Subtotal
11	m	Rectum	Rectum carcinoma	Radio-chemotherapy	993	47	Radiotherapy	Cranio-cervical junction- C2	IM	Weakness in right arm, paresthesia	Lung, bone	Yes	No	Improved motor and sensory function, ambulating independently	Gross-total
12	m	Prostate	Paraparetic syndrome	Radio-chemotherapy	303	68	Palliative radiotherapy	T 8	IM	Reduction in strength below pelvic girdle, not ambulatory	Bone, lung, lymph nodes	No	Yes	Minor improvement in motor function, not ambulatory	Subtotal
13	f	Lung (small cell)	Cerebellar metastasis	No therapy	445	68	Palliative treatment	T 2–3	IM	Discrete paresis of right leg, hypoesthesia right leg, ataxia	Lung, lymph nodes	Yes	No	Improved motor function, ataxia unchanged compared to preoperative status	Gross-total

**Table 3:** Surgical characteristics and postoperative outcome *.

Characteristics	(*n* = 13)
surgical approach, no. (%)	
hemilaminectomy	3 (21.1)
laminectomy/laminoplasty	9 (69.2)
staged anterior + posterior stabilization and tumor resection	1 (7.7)
mean operation time, min.	181 ± 92
resection rate, no. (%)	
gross total resection	7 (53.8)
subtotal resection	6 (46.2)
biopsy only	0 (0.0)
median length of in-patient stay, days (range)	12 (6–71)
postoperative surgical complications, no. (%)	
CSF fistula	0 (0.0)
hematoma	0 (0.0)
wound breakdown	0 (0.0)
infection	0 (0.0)
postoperative change in neurological status, no. (%)	
improved	8 (61.5)
stable	4 (30.8)
deteriorated	1 (7.7)
postoperative McCormick score, no. (%)	
I	2 (15.4)
II	3 (21.1)
III	3 (21.1)
IV	5 (38.4)
postoperative change in McCormick score, no. (%)	
improved	6 (46.2)
stable	6 (46.1)
deteriorated	1 (7.7)
adjuvant treatment, no. (%)	
radiotherapy	9 (69.2)
chemotherapy/targeted therapy	4 (30.8)
local recurrence rate, no. (%)	2 (15.4)
median time period until local recurrence, months (range)	12 (6–17)
reoperation rate for local recurrence during follow-up, no. (%)	1 (7.7)

* Mean values are presented ± standard deviation.

**Table 4:** Preoperative risk factor analysis affecting survival after surgery for intradural spinal metastasis.

Univariate	Hazard Ratio (*p* Value/95% CI)
age at surgery	
per year	1.01 (0.80/0.97–1.04)
preoperative KPS	
score value	0.99 (0.64/0.94–1.04)
preoperative McCormick score	
I vs. II vs. III vs. IV	1.81 (0.17/0.77–4.22)
multiple systemic metastases (at the timepoint of surgery for intradural metastasis)	
yes vs. no	1.99 (0.38/0.43–9.32)
additional central nervous system metastases (at the timepoint of surgery for intradural metastasis)	
yes vs. no	0.99 (0.98/0.31–3.15)
relationship to spinal cord	
completely extramedullary vs. at least partly intramedullary	1.01 (0.98/0.32–3.48)
location	
cervical vs. thoracic vs. lumbar	3.27 (0.02/1.21–8.87)
extent of tumor	
per level	0.93 (0.89/0.30–2.84)
Multivariate	Hazard Ratio (*p* Value/95% CI)
age at surgery	
per year	0.97 (0.41/0.90–1.05)
preoperative KPS	
score value	1.03 (0.56/0.94–1.11)
preoperative McCormick score	
I vs. II vs. III vs. IV	8.16 (0.04/1.09–61.31)
multiple systemic metastases (at the timepoint of surgery for intradural metastasis)	
yes vs. no	6.03 (0.28/0.24–152.94)
additional central nervous system metastases (at the timepoint of surgery for intradural metastasis)	
yes vs. no	5.47 (0.20/0.40–74.30)
relationship to spinal cord	
completely extramedullary vs. at least partly intramedullary	0.29 (0.29/0.03–2.84)
location	
cervical vs. thoracic vs. lumbar	15.11 (0.02/1.44–158.97)
extent of tumor	
per level	0.36 (0.52/0.02–7.38)

OR: odds ratio, CI: 95% confidence interval.

**Table 5:** Case series reporting on survival outcomes of surgically treated intradural spinal metastases.

Study	Year of Publication	No. of Cases	Extramedullary /Intramedullary Spinal Metastases	Mean Survival (after Surgery)	Minimum Length of Follow-Up	Percentage of Censored Data
Chow et al. [1]	1996	10	extramedullary	10.7 months	3 months	40%
Wostrack et al. [4]	2012	9	4 intramedullary, 5 extramedullary	7.3 months	3.5 months	11%
Hoover et al. [9]	2012	15	3 intramedullary, 12 extramedullary	5 months	n.a.	33%
Sung et al. [34]	2013	8	intramedullary	4.5 months	1 month	-
Payer et al. [8]	2015	22	intramedullary	11.6 months	2 months	50%
Goyal et al. [35]	2019	8	intramedullary	4.5 months	2 months	-
Gazzeri et al. [6]	2021	43	extramedullary	9.6 months	5 months	16%
Wu et al. [33]	2022	6	intramedullary	5 months	<1 month	50%
Kritikos et al. [23]	2024	9	6 intramedullary, 3 intra/extramedullary	7 months	5 months	44%

## Data Availability

Data available on request due to restrictions because of legal and ethical reasons.
